# Visual memory

**DOI:** 10.3758/s13414-025-03214-3

**Published:** 2026-02-23

**Authors:** Geoffrey F. Woodman, Sean M. Polyn

**Affiliations:** Department of Psychology, Vanderbilt Vision Research Center, Vanderbilt University, 2301 Vanderbilt Place, PMB 407817, Nashville, TN 37240-7817 USA

**Keywords:** Visual working memory, Memory: Long-term memory, Visual memory and face recognition

## Abstract

Visual memory allows us to behave adaptively in the world we live. In this tutorial we will review the types of visual memory storage that have been identified. These storage processes begin the instant a visual stimulus appears and continue through to remembering objects and scenes that were encountered decades ago. The different types of memory storage have different properties of capacity and resolution. We will discuss how our memories allow us to link new information to information that we have acquired across our life spans. We will also discuss how this linking between new information and previously acquired visual information is an active process, in which memories shape how we interpret and store new visual inputs.

## Introduction

**Fig. 1:**
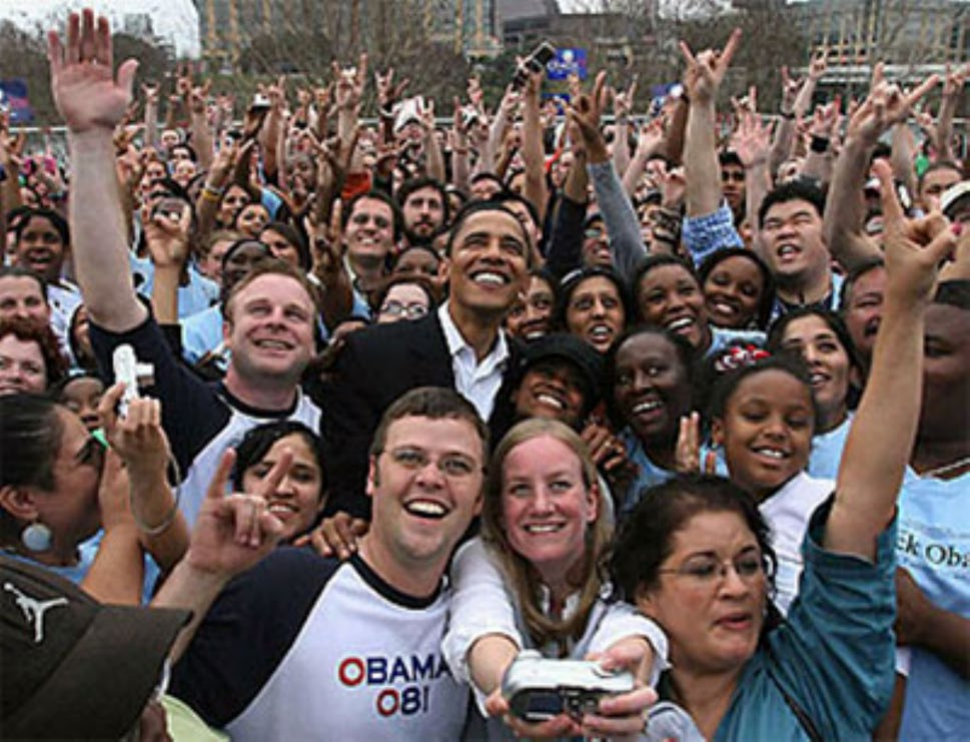
A photograph of a real-world scene that illustrates the nature of different types of memory storage described in this tutorial. If you look at the picture and close your eyes, you can experience the image fade within 250–500 ms. After this initial fading, you can probably remember about three details of the scene (i.e., three faces that you saw in the crowd). If you close the article, and take a walk, you will probably be able to recall the gist of this picture from memory if a friend asks you what you were studying

We take memory for granted. In our typical papers in *Attention Perception & Psychophysics*, we describe experiments in which the visual system is exposed to a stimulus, and then the observer reports what she sees (she is a human, monkey, cat, or even a neuron). For example, when we see a simple object, like a rightward-tilted red bar, what makes it a rightward-tilted red bar? Clearly, the ability of our visual system to discriminate this stimulus is based on our experience seeing colors and bars in the past. Our visual systems do much heavier lifting in the real world. Look at the picture in Fig. 1. Within a couple of hundred ms of glancing at this picture you will have been able to say that it is a picture of an election event. You can recognize a past President of the United States and determine that you do not remember anyone else from that picture.

Since its inception, memory research has been dominated by experiments using verbal stimuli (i.e., letters, words, sentences; see Palmer, 1999). As a result, our discussion of memory will present the principles of memory storage that have been derived from experiments using a variety of stimuli, but mostly verbal materials. We will then discuss how memory may differ for stimuli that can be verbalized versus stimuli more typical of vision science, when such details are known.

Experiments have shown that our memories change across time. Specifically, it appears that our visual systems initial store a huge amount of information, but this type of immediate memory storage, known as iconic memory, is very fleeting (Sperling, 1960). It appears to fade within 250–500 ms. Next, it seems that we can store a few pieces of information in a more durable fashion. This is what memory researchers call short-term memory or working memory (Luck & Vogel, 2013). The latter term, *working memory*, emphasizes our ability to use these few pieces of information to perform tasks and manipulate this information in our heads (Baddeley, 2007). Finally, some of the information that we store across the short term lays down a durable enough trace to become a usable long-term memory (Hollingworth & Henderson, 2002). By usable, we mean that we can retrieve that information minutes, hours, or even years later (Nobel & Shiffrin, 2001; Shiffrin & Atkinson, 1969). As we will also discuss later in this article, information can be stored in long-term memory that influences subsequent behavior even though we are not aware that we have stored it in memory. This is known as implicit memory (Chun & Jiang, 1998; Reber, 2013; Schacter, 1987), and we will discuss several types of such effects.

**Fig. 2:**
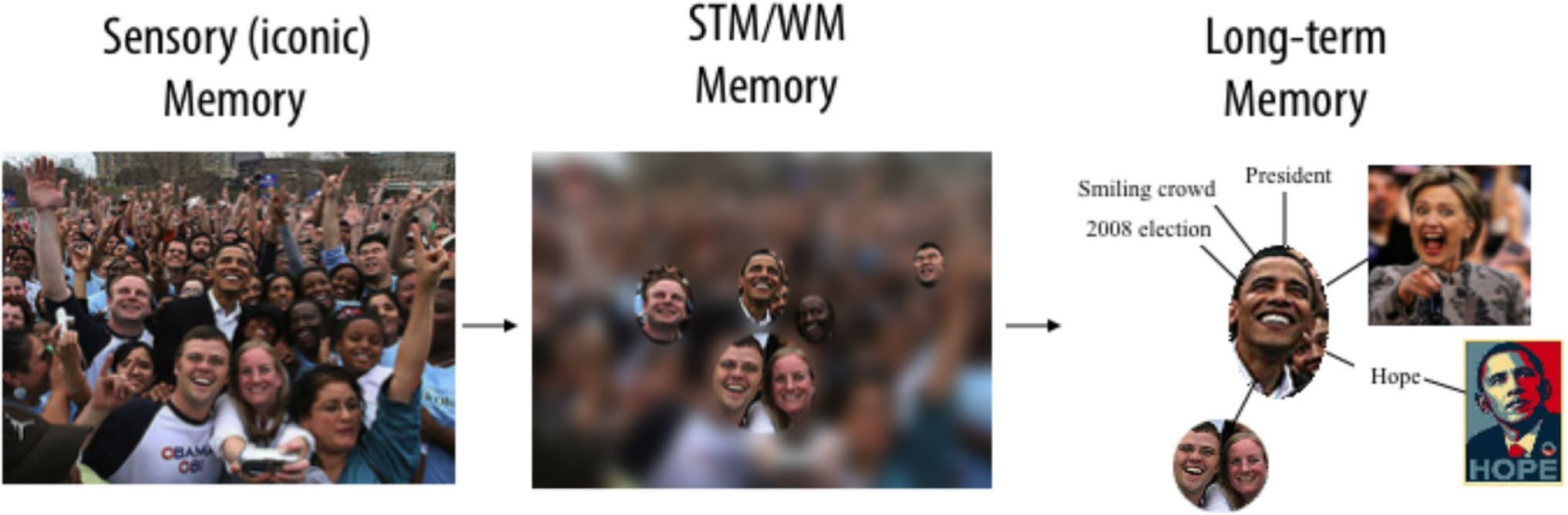
A schematic representation of the nature of different types of memory storage proposed in the modal model of memory (Atkinson & Shiffrin, 1968). Research suggests that when information enters the visual system it is first stored in iconic memory (on the left) that has a large capacity and is very precise but fades very rapidly. Next, some of the information can be encoded into visual working memory. Only about three objects worth of information can be stored in visual working memory, but these can be maintained and manipulated in this limited-capacity store. Some information from the scene in Fig. 1 will be encoded into long-term memory, where it will be associated with existing memory representations of other episodes and linked to our general semantic memory

The idea that memory storage passes information through distinct stages is characterized in Fig. 2. The idea that visual information is first stored in iconic memory, then short-term memory, then long-term memory became the focus of research in the middle of the last century (Atkinson & Shiffrin, 1968). This model of memory has received extensive criticism (Plancher & Barrouillet, 2020), such as evidence that information that does not make it into working memory can influence long-term memory (Marcel, 1983). However, it continues to account for a substantial amount of data from laboratory experiments and guides scientific thinking about how we store the visual information that our visual systems process (Wixted, 2024). Our first topic of discussion is how information is stored immediately after it activates the visual system.

### Iconic memory

When a stimulus is presented and then disappears it leaves a trace of activity in our visual system that fades quickly. You can easily demonstrate this effect to yourself by closing your eyes and observing the scene you were viewing rapidly fade. This perceptible fading of the visual world has been known as visual persistence (Di Lollo & Dixon, 1988). It was initially noted that an ember swung through the air on the end of a stick would leave a trail on a dark night. As the study of vision grew into a science, this was studied more carefully (Di Lollo, 1980; Di Lollo & Dixon, 1988). Much of the still-definitive work on the nature of this immediate and fast decaying type of memory storage was performed in the 1960 s, as we discuss next.

**Fig. 3:**
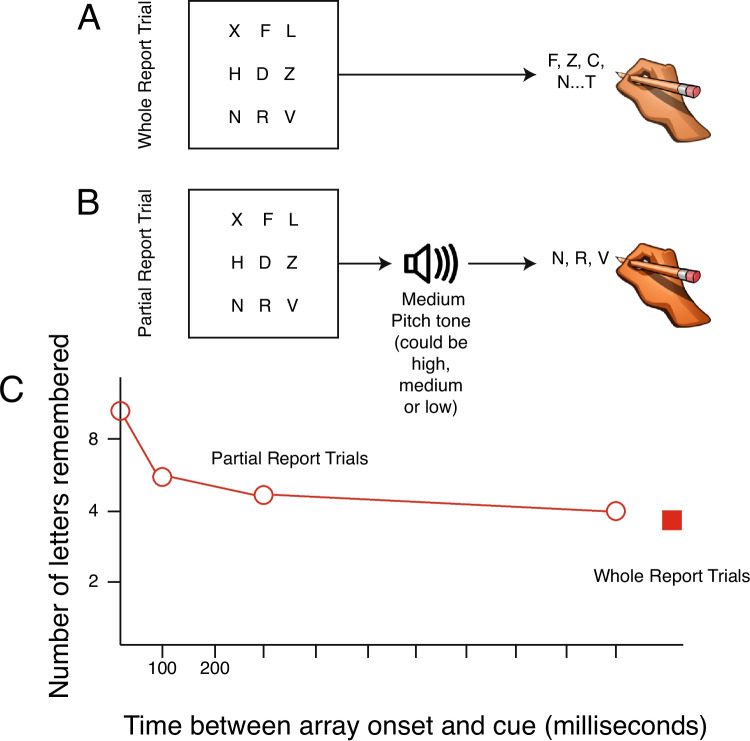
The task and results of experiments performed by Sperling (1960) to measure the storage of information in iconic memory and the rate of information transfer from iconic memory into short-term memory. **A** An example whole-report trial in which the subjects need to remember all of the stimuli from the array that is flashed for 50 ms. Then, subjects wrote down as many letters as they could remember from the entire array. **B** An example of a partial-report trial in which the pitch of a tone indicated which third of the array to report: the top, middle, or bottom line of letters.** C** The data from Experiment 4 of Sperling (1960) plotted showing the decay of the information across time on the *x*-axis. The *y*-axis plots performance of the number of letters remembered on each trial (out of 3) and multiplies it by the number of rows of letters that were available to apprehend (3 rows), so reporting 2 letters of the 3 in the upper row would equal 6 letters available to report in memory

By quickly flashing an array of letters or shapes, Sperling (1960) and Averbach and Coriell (1961) showed that people initially had access to all of the information in the large arrays they presented. But this information rapidly decayed as time passed following the 50-ms presentation of the arrays. Figure 3 shows an example of a stimulus array from one of these experiments. The task involved either reporting all the items in the array (called *whole report*; see Fig. 3A) or just the items in the stimulus arrays that were signaled by a cue (called *partial report*; see Fig. 3B). For example, on partial report trials in which a high tone was presented, people were supposed to report the items in the top row of the array. When the cue was presented at the same time as the stimulus array, or very shortly thereafter, people could report all of the stimuli in the row that the cue indicated. By adding together the number of letters remembered when the top, middle, and bottom rows were cued, we can see that people could remember almost all nine letter in the array (i.e., the estimate of the number of letters remembered). However, when the cue was presented at longer and longer intervals after the brief array of items appeared, the people had access to fewer and fewer items. Ultimately, the function in Fig. 3C shows that accuracy reached asymptote equivalent to three to four of the letters, just like people could report on whole report trials. These findings were interpreted as providing evidence that we initially have access to a type of memory storage that is very high capacity, in that it appears to store essentially everything we see; however, it is very delicate, in that is decays rapidly and is gone quickly (Coltheart, 1980). This type of visual memory storage is known as iconic memory.

*Iconic memory* is the term for our fleeting visual memories that fade within 250–300 ms of the disappearance of a stimulus (Coltheart, 1980). Other modalities seems to have similar *sensory memory stores*, with echoic memory being the memory store for auditory information that appears to last longer than iconic memory, up to 10 s based on some estimates (Lu et al., 1992). Iconic memory is fragile, in that it can be wiped out by the presentation of another stimulus (i.e., visual masking), or by an eye movement (G. R. Loftus et al., 1985). Due to the fragile nature of the memories in iconic memory, it appears that if we want to remember a visual stimulus for more than a fraction of a second, we need to encode it into a more durable type of memory store (Gegenfurtner & Sperling, 1993).

### Visual short-term memory and working memory

**Fig. 4:**
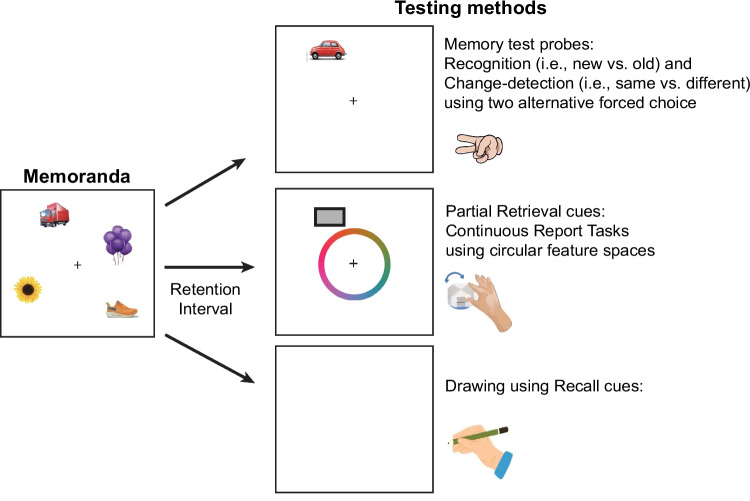
Example of the tasks used to test visual memory in general. The left panel shows an example of a stimulus array used to test subject’s memory for common objects across short retention intervals of less than a few seconds in working memory tasks (typically change-detection tasks) and across longer intervals in long-term memory tasks (typically involving remember objects or scenes across fill distraction intervals or the study of additional objects in recognition memory testing). Continuous-report tasks can be used to probe memory across the short or long term provide measures of how precisely people remember the feature of an object, typically responding using a spatial response on a circular representation of feature space (such as the color wheel used here in which subject click when the cursor is on top of the color they remember the object at that location being). Finally, the bottom right panel depicts the use of drawing tasks to provide a visual analog to recall task from the long-term memory literature (Bainbridge et al., 2019)

Few representations that are temporarily buffered in iconic memory can be encoded into visual short-term memory so that we can continue to remember them after an object or a scene disappears from view. This has been shown using a simple *change-detection task* (Phillips, 1974; Vogel et al., 2001). In this task, subjects view a memory sample array, followed by a brief retention interval, and then a test array (see Fig. 4). Subjects’ task is to determine whether any of the objects change between the sample and test array. Using this type of task, researchers showed that the average human subject can remember about three simple objects (Luck & Vogel, 1997, 2013; Vogel et al., 2001). Models have been proposed to account for this extreme capacity limit appealing to concepts of slot-like object representations (Rouder et al., 2011), the allocation of infinitely divisible neural resources (Bays et al., 2009), as well as combinations of these capacity limiting mechanisms (Zhang & Luck, 2008). In addition, feature-based storage structures have been proposed (Treisman & Zhang, 2006; Wheeler & Treisman, 2002). Regardless of the representational substrate, these representations are more durable than iconic memory and can be held in short-term memory to perform tasks such as imagining a map after viewing it. Demonstrating how this real-world use of memory representations is possible, laboratory experiments have shown that after a representation of an object is encoded into short-term memory, it appears to be largely immune to masking by a new visual stimulus (Vogel et al., 2006; Woodman & Vogel, 2008); however, it can be displaced by new information that is encoded into short-term memory (Parmentier et al., 2004). Most importantly, we can use the representations in short-memory memory to perform work (Baddeley, 2003). This resulted in the term *visual working memory* becoming the dominant model of how this type of short-term memory storage functions.

The idea that our visual short-term memory is really a visual working memory was proposed to account for how we can perform tasks like mental arithmetic (Baddeley, 2007). This is probably one of the primary ways that we all use visual working memory on a daily basis. What is 6% of $320,060? When performing complex multiplication or division in which we do not have the answer memorized, we frequently imagine seeing the numbers in our heads (i.e., we imagine placing the 5, carrying the 2, etc.). However, many professions heavily use visual working memory as the representational workspace to imagine spatial layouts (architects, scientists, carpenters, etc.).

A substantial amount of research has focused on the nature of the representations in visual working memory. Early research showed that the representations of complex objects were essentially independent of spatial location (Phillips, 1974). In the past 20 years, the debate about the nature of the representations that we temporarily store and manipulate has been focused on whether these are object representations (Luck & Vogel, 2013), or whether people can represent a limited amount of information spread across virtually any number of inputs (Bays & Husain, 2008). This debate has set up competing models in which people propose that there are a limited number of object slots in visual working memory, or that visual working memory is a limited resource that is spread across either a few objects (that can be remembered very well) or many objects (with each of that large number of objects remembered poorly). Data from continuous-report tasks, in which participants click on a color wheel to report the color of an object in memory, have played a central role in this debate (Bays et al., 2009; McMaster et al., 2022; Smith, 2016; Zhang & Luck, 2008).

Although the theoretical debate about the nature of the limited capacity of visual working memory is still unfolding, several features of visual working memory storage are clear (Oberauer et al., 2018). First, visual working memory has a capacity limit that is much smaller than iconic memory. Second, the representations of visual working memory are more durable and longer lasting than the fast-decaying representations in iconic memory. Third, as we found with the cues used in Sperling (1960), it appears that attentional selection helps or is even required to gate what information gets into visual working memory from visual perception and iconic memory (Vogel et al., 2005). A full discussion of the relationship between attentional selection and working memory storage is beyond the scope of this review; however, it is sufficient to note that the attentional blink (Chun & Potter, 1995; Shapiro et al., 1997) and inattentional blindness research support the conclusions that attentional selection is necessary for encoding into visual working memory and awareness (Most et al., 2005). Our discussion brings us to the final type of memory representation in the modal view of memory—that is, long-term memory.

### Long-term memory

According to classic ideas in the study of memory, long-term memory is the name for representations that are maintained in the visual system in a more-or-less permanent fashion. Long-term memory is amazingly large. There appears to be no limit to the amount of information our visual systems can store in long-term memory. A classic study by Lionel Standing had people view 10,000 pictures from magazines for several seconds each, across 3 days (Standing, 1973). Then, the subjects had their memory tested for those precise pictures the next day, 3 days later, and a week later. He found that memory for the visual details of these pictures was highly accurate (i.e., better than 90% correct in recognition testing even after a week). So, people can remember a huge number of visual images or scenes. An implication is that these vast reserves of long-term memories can be drawn upon to support working memory in a wide variety of tasks involving skilled performance, including chess, music performance, and even cognitive tasks like digit span (Ericsson & Kintsch, 1995).

**Fig. 5:**
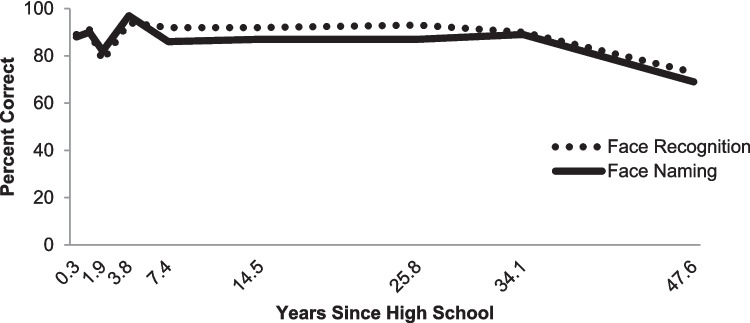
The extremely long-term memory results from the cross-sectional study of Bahrick et al. (1975). The accuracy of people's memory for people they went to high school with. The face recognition test asked the people to determine whether the face was or was not someone from their class. The face-naming task required them to name the person shown (either first or last name counted as correct. These scores correct for attendance at reunions, whether people looked at their yearbook, had kept in contact with high school friends, et cetera. Note that people who are over 50 years of age, and have not seen these people since high school, continue to perform as well as recent graduates

We can remember at least some of visual images across our entire life. Bahrick and colleagues ran a now classic study showing that even 50 years after graduating from high school (see Fig. 5), people can still recognize almost all of their classmates’ randomly selected pictures from their high-school yearbook versus similar photographs from other yearbooks (Bahrick et al., 1975). Note that this incredible duration of the memories is working against the general cognitive decline that the participants experienced with normal aging. Later we will discuss why it is that we forget, but it is important to keep in mind that whereas iconic memory was limited to a couple of hundred ms, and visual working memory was limited to just a few objects worth of information, visual long-term memory has no such limits in either time or quantity.

### The early days of memory research

We cannot discuss what is known about memory without highlighting Hermann Ebbinghaus (1885/1913). To study memory, Ebbinghaus invented his own stimuli that were called the trigrams. These are artificial stimuli that were designed so that subjects would not already be familiar with them, allowing Ebbinghaus to study the process of learning novel stimuli. These stimuli were the precursors to the Gabor patches (Danker et al., 2008) and greebles (Gauthier et al., 1998) currently used to study responses in visual cortex and processing of novel visual stimuli. The trigrams were strings consisting of a consonant-vowel-consonant that did not appear in the language, in the case of Ebbinghaus, that language was German. His experiments are interesting not only because he created a new kind of stimuli for memory experiments that had never been conducted, but also because of his primary subject.

He was the subject of his experiments. He would randomly create lists of trigrams (e.g., TET, WEZ, HUR) and study them until he would recite them from memory perfectly (i.e., 100 % correct recall of the list). He would then test himself at different times after he had perfectly learned these lists.

**Fig. 6:**
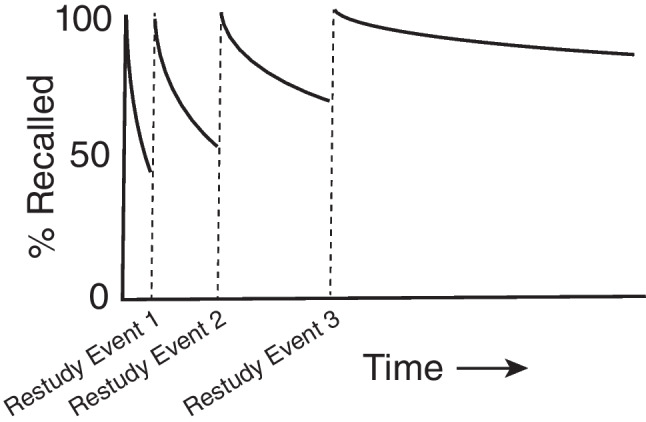
Idealized plots of the rate of forgetting following the learning of random strings of three letters based on Ebbinghaus (1885/1913). We forget information more slowly the more we have studied it

Two critical observations were first made in these experiments of Ebbinghaus and are shown in Fig. 6. The first observation is that after he learned a given list of trigrams, he forgot the information from the list at a predictable rate. The bad news is that the rate of forgetting is initially very steep. That is, you and I very rapidly forget information right after we learn it if we do not make an effort to remember that information. Across a large number of lists, Ebbinghaus forgot about half (50%) of each list within 1 hour after learning it. The good news is that the forgetting leveled off after this initial steep part of the curve, such that after about 2 days, Ebbinghaus could remember about a fifth of the words from a list, and this was still the case after about 20 days. The forgetting curves in experiments like these can be accurately modeled with a power function (Wixted, 2022), or exponential function (Heathcote et al., 2000). Anderson and Schooler (1991) argue that these mathematical functions may be optimized for human experience, in that they seem to capture the likelihood that a piece of information will be needed again, given how often it has occurred in recent naturalistic experience. The second major observation that Ebbinghaus made was that the forgetting following the first episode of learning could be arrested by restudying the list. This is shown in Fig. 6 with the restudying the list. Restudy of the artificial trigrams returned immediate memory test performance to 100% correct. However, it also changed the nature of the forgetting functions. With each episode of restudy, the forgetting function would decrease less rapidly. This means that by the third or fourth repetition of the same list, forgetting was much more gradual than following a single study episode.

This is great news for students. Ebbinghaus’ experiments showed that one way to learn material is to study it over and over again. However, as we will discuss later in this article, there are more efficient methods to learn material, and more complex visual material, like scenes, faces, or diagrams may have forgetting functions that are naturally less steep than verbal materials, such as words or the trigrams that Ebbinghaus used (Hollingworth & Henderson, 2002).

If every time we restudy a set of material, such as a list of trigrams, we are encoding that information back into visual working memory, then that will improve the quality of our long-term memory representations of that stuff (Logan, 2002). This can explain why restudy improves our retention of information in long-term memory. But why does forgetting happen at all?

### What causes forgetting?

**Fig. 7:**
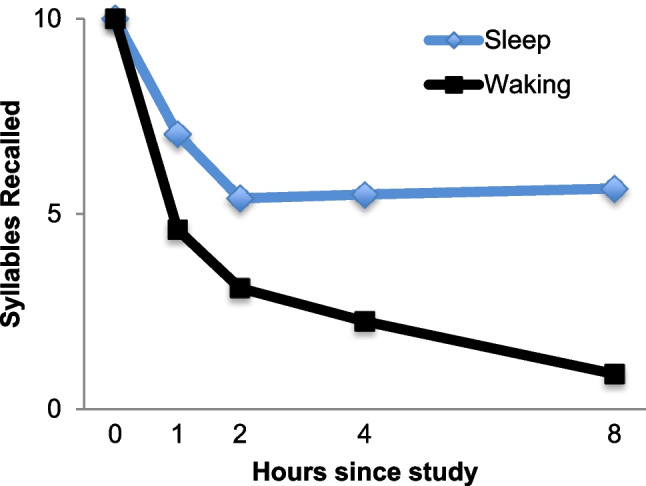
Results from Table III in Jenkins and Dallenbach (1924) showing that forgetting is slowed by sleep

There have been several proposal for why we forget. One proposal is that memories decay (Pratt, 1936). The idea is that memories simply break down as time passes. There is an intuitive appeal of proposing that memories are like essentially every material in the physical world. Indeed, the forgetting curves look like radioactive decay functions (see Figs. 6 and 7). If this idea is correct, then once we have formed a shiny new memory, it will fall apart quickly, and then more slowly, even if nothing else happens. Fortunately for our memories, decay does not appear to happen.

It appears that if we can stop experiencing new events, then forgetting slows or even stops. Figure 7 shows how this was tested in an experiment in which students were either awake or asleep during a memory retention interval (Jenkins & Dallenbach, 1924). After falling asleep, no additional forgetting was measured. In contrast, in the waking condition forgetting could be measured across time, just as Ebbinghaus observed. If a memory were breaking down across time it should not matter what other things the brain is doing. But instead, it appears that simply being awake and having our brain experiencing new things results in forgetting (McGeoch, 1932).

Consistent with the idea that forgetting is caused by the accumulation of new information in the brain, another study taught cockroaches to avoid a region in their environment (Minami & Dallenbach, 1946). Then, during a 24-hour interval following learning, the cockroaches were either allowed to run around in their home environment or held inactive in a paper cone. The cockroaches in the paper cones that were prevented from experiencing new things forgot significantly less than those that continued living their cockroach lives and experiencing new things. These findings suggest that we forget because we experience new events, either as we move through the environment, like the cockroaches, or because our minds continue to imagine future events or relive recent episodes. Consistent with this proposal, when cockroaches are forced to engage in more activity than typical, to elicit a richer set of experiences that could interfere with memory, then even more forgetting was observed.

The idea that learning new stuff causes the forgetting of things we have previously learned attributes forgetting to *interference* (Melton, 1963). That is, after having witnessed an event, like a crime, we then have new experiences and see new things. These new things lay down long-term memory traces of their own that overlap with our old representations of the crime scene. Unlike the difficulty researchers have had finding evidence for memory decay, interference between memory representations can be found in virtually every study that has looked for it.

The best-controlled experiments that have studied interference between information in memory have used a paired-associates paradigm (Roediger & Schmidt, 1980). This involves teaching people new, arbitrary pairs of items. For example, I ask you to remember frog-41, bathtub-18, wristwatch-92, and so forth, until I can then ask you to tell me what is paired with each associate (i.e., what goes with bathtub?) at a criterion level (i.e., 90% of the time). People can do this accurately after a bit of study with a list of associates. However, if I then have you learn a new list composed of frog-75, bathtub-52, wristwatch-12, and so forth, then learning these new associates interferes with your ability to remember the associates from the first list. You can still get a reasonable number correct from the first list of associates, so it is not as if the new list overwrites the old one. But inference can be measured with a reduced accuracy compared to conditions in which the same amount of time passes without learning a new list or learning a list that contains no overlapping pairs (i.e., frog, bathtub, and wristwatch are in both lists in the interference condition, but in the comparison condition rabbit, table, and hat are paired). We can also measure *intrusion errors*, these are errors in which you report pairs from the new list, believing that you remember them from the first list (Davis et al., 2008) or array of visual items (Zhou et al., 2023). These errors have been useful in making inferences about how we select information from memory, and it appears the same forces that cause interference in these verbal tasks are at play in visual memory tasks as well (Oberauer & Lin, 2017, 2023).

It is important for us to highlight one aspect of the experiments we have just been discussing. Specifically, these experiments used lists of stimuli, with the exceptions that we noted. This use of lists of memory items is meant to simulate the continuous flow of new information that we experience as we move through life (Manning, 2021; Tulving, 2002). In both cases, something happens and then something else happens. Thus, many researchers focused on episodic memory analyze memory performance by examining memory across items in a list, and then across lists, to understand how the brain stores our constant flow of visual information as we move through each day. Obviously, lists of words are a poor approximation for the seemingly continuous flow of our visual life; however, the principles that govern memory for the two types of information have not been shown to be radically different so far (Surprenant & Neath, 2009).

### False memory

Frequently we have the intuition that our memory for visual information is immune to interference or contamination by new information that we encounter after witnessing an event or viewing a stimulus. However, it appears that our memories are malleable and can be changed even after they are initially encoded. At the extreme, this interference creates what researchers call *false memory*. False memory is when a subject reports remembering something that did not happen or inaccurately remembers the details of an event that was witnessed. In a classic set of experiments on false memory, researchers had observers view a series of films depicting car crashes (E. F. Loftus & Palmer, 1974). The observers were then asked questions about each car crash and were randomly assigned to different conditions in which the questions were asked using slightly different wordings. When asked about the speed of the cars during the accident when the cars “contacted” each other, observers estimated that the cars were going 32 miles per hour (mph). When the researchers used the word “hit,” the estimate was 34 mph. It was 38 mph using the word “bumped,” 39 mph using the word “collided,” and 41 mph using the word “smashed.” The questions that immediately followed each film had long-lasting effects on people’s visual memory, affecting visual details they would report a week later during a follow-up session. About 7% of observers later reported seeing broken glass on the street after the crash when they were initially asked how fast the cars were going when they “hit” each other. But when the initial question used the words “smashed into” the reports of broken glass doubled to 16%. Follow-up work has raised questions about the reliability and replicability of this finding (Goldschmied et al., 2017). However, as we review below, other work has identified more reliable ways to examine false memory using visual materials.

**Fig. 8:**
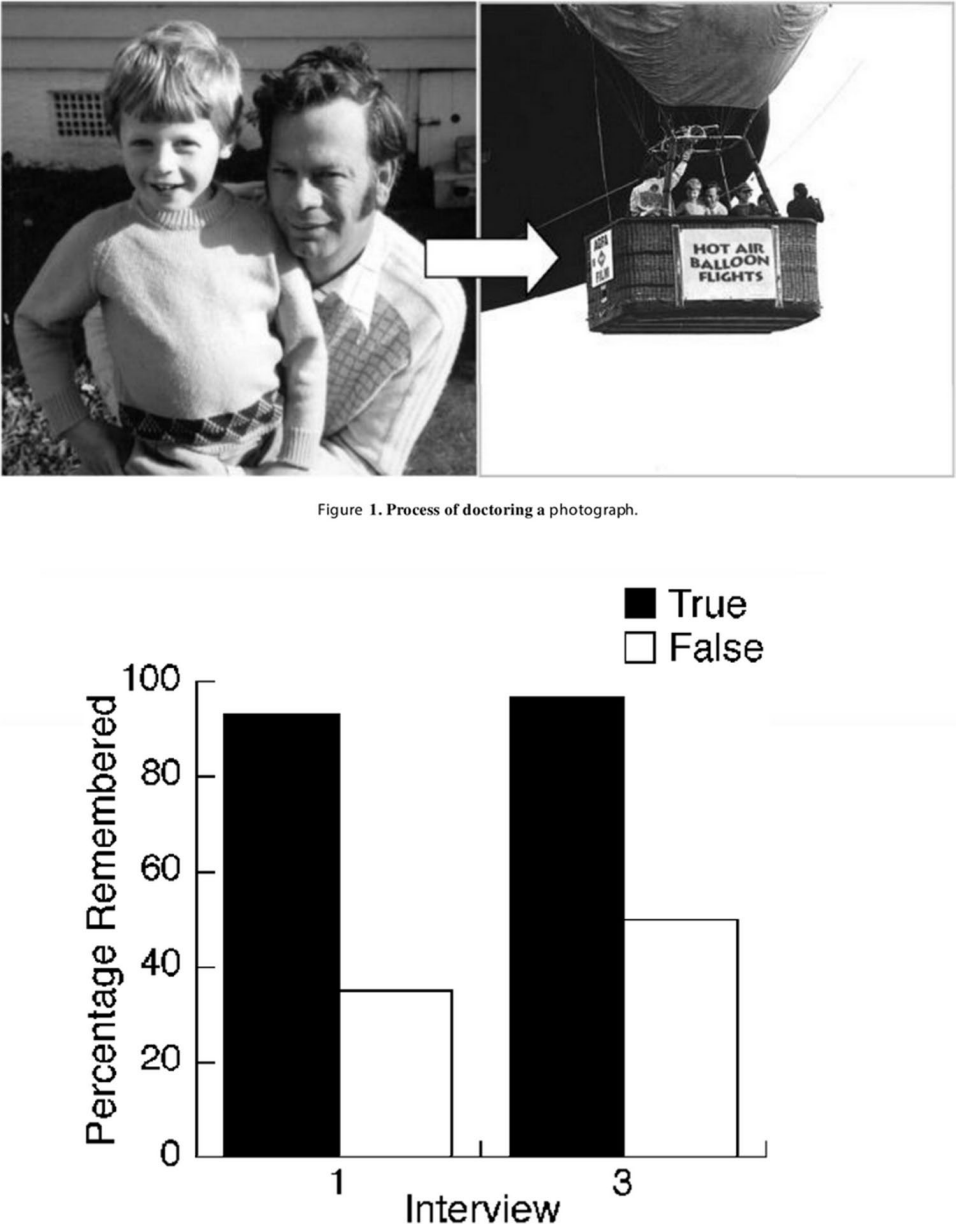
Illustration of the stimuli and results of Wade et al. (2002). Adapted with permission

It appears that visual input can cause extremely strong interference on our memories. One such study of false memory showed how pictures can have a particularly strong influence on how we remember events, even from our own lives. Wade et al. (2002) doctored photographs to make it appear that the subjects in their memory experiments had taken a hot-air balloon ride as a child. The experiment involved enlisting adult members of the subject’s family to verify that they had not taken a balloon ride as a child, and to provide photographs from the subject’s childhood so that stimuli could be created for their experiment. During the first interview, subjects were asked if they remembered the event in the doctored photograph in which their picture had been Photoshopped into the picture of a hot-air balloon ride (see Fig. 8). By the time of the third interview, 1–2 weeks after the first interview, the number of subjects remembering the balloon ride event had doubled, with many of these providing vivid details from the now-remembered event. Given the increasing availability of software for the manipulation of pictures, these laboratory findings are troubling.

We might not be surprised that getting misleading information is sufficient to interfere with the information that we have already stored in long-term memory. Indeed, many memory researchers view this as adaptive in most circumstances. We need to be able to integrate and learn from new information. It would be unwise if we continued to view a neighbor’s dog as a stimulus that we should approach to pet if it bit us yesterday. However, there are other phenomena that have been identified in the laboratory that suggest our memories can be degraded simply by accessing a related memory.

Researchers have shown that if you remember one piece of information about a scene or an event, it appears to cause the forgetting of other information stored about that scene or event. In the laboratory, these effects are known as retrieval-induced forgetting (Anderson et al., 1994) and recognition-induced forgetting (Maxcey & Woodman, 2014), depending on the type of test used to probe memory. These findings indicate that simply remembering an object that we saw earlier today, or yesterday, may result in the forgetting of other objects that we saw at a similar time. Again, this is likely adaptive, assuming the objects that we frequently remember are the most important ones, but it can make our lives difficult when we desire to remember everything we see at an event (e.g., a crime that we witness, or an important athletic event) instead of just the information that we are later asked to recall by someone we are telling about the event.

In summary, even after we store visual representations into long-term memory, postevent information can have an enduring effect on the nature of these memories. These findings from the laboratory have implications for how we use eye-witness reports in legal settings, as well as whether we can completely trust our own memories for visual details from our lives.

### Flashbulb memories and the modulation of visual memory by emotion

Certain memories appear to be recalled from visual long-term memory with particularly vivid detail. These are known as *flashbulb memories* because observers report remembering them with such detail that it is as if their visual system took a high-resolution photograph of the scene, assisted by the flashbulb of a camera (Hirst et al., 2015). Usually, these are events that elicit strong emotional reactions in the observer. For example, the birth of a child, a car crash, scoring a winning goal in a game, the assassination of a president, or some other event that is particularly wonderful or terrifying. It appears that the brain mechanisms responsible for our emotional experiences interact with the basic cognitive machinery responsible for memory to encode memory representations more accurately.

Moments of national tragedy in the United States have been central in these studies (e.g., President Reagan’s assassination attempt; Pillemer, 1984), the space shuttle *Challenger* explosion (Bohannon, 1988), and the terrorist attacks of September 11, 2001 (Hirst et al., 2015; Talarico & Rubin, 2003). The last of these was studied by testing the memories of students from Duke University for details of the terrorist attacks (e.g., “Were others present when you first heard the news?”) compared with other details of ordinary events from that day (“Were there others present when you studied for class that day?”; Talarico & Rubin, 2003). The students then had their memories tested for the flashbulb event and the ordinary events after 1 week, 6 weeks, and almost 1 year. People’s flashbulb memories about the terrorist attack events were no more accurate than memory for ordinary events. In addition, they forgot the details of both types of events at the same rate across time.

What did differ was people’s confidence about the accuracy of their memories. People thought that their memories for the events surrounding the terrorist attacks were highly accurate, even though accuracy was slightly higher for the ordinary events. In addition, this study had subjects rate their initial visceral emotional reaction to the terrorist attacks. These initial ratings were unrelated to memory accuracy but did predict how overconfident the subjects were in the details of the flashbulb events later in time. Thus, these flashbulb memories that we believe to be highly detailed visual representations of an emotionally charged event do not appear to be different than our memory for other highly ordinary, high-frequency types of events, such as eating dinner, other than our strong beliefs that these memories are special.

### Visual long-term memory is better than memory for other information

The attentive reader will remember that we began this article discussing the fact that the majority of memory research has been done while requiring subjects to remember verbal materials. Although the principles of remembering and forgetting appear to be very similar whether human subjects are remembering syllables and words, or objects and scenes, there are some important differences that have been identified. The most important difference is that memory for visual information is better than memory for verbal information.

Previously, we discussed the study of Standing (1973), showing that people could remember a huge number of photographs across days. This study directly compared the ability of human subjects to remember photographs compared with words. He found that people remembered the photographs significantly better than the words, even though the time provided to learn them was equated. For example, from 1,000 photographs of *vivid* *events*, such as a place crash, a dog smoking a pipe, and so on, subjects remembered 880. Subjects remembered 770 of the 1,000 normal photographs of a plane, a dog, and so on. However, only 615 of the 1,000 words were remembered when 5 seconds with each type of stimulus was given to study. This superiority of memory for visual information over verbal information has been observed countless times since, using a variety of different stimuli and different tasks (Brady et al., 2008; Hockley, 2008).

There have been different hypotheses proposed for why visual memory is superior to verbal memory. One proposal is that when people see a picture of an object they spontaneously name that object, resulting in dual coding of that stimulus in memory (Paivio, 1976). Then, when it is time to retrieve information about that picture, there is both the visual and verbal representation that have been encoded into memory, doubling the chances that we can use one of these two retrieval cues and remember what we saw. Others have hypothesized that visual stimuli, such as pictures, benefit from automatically receiving more extensive semantic analysis, that is, people extract the hierarchical meaning of a picture when they might not do so with a word (Weldon et al., 1989). This is important because classic studies have shown that when people focus on the meaning of a stimulus, they remember it better than when they simply focus on its surface features (Craik & Lockhart, 1972). Yet another account is that pictures and visual stimuli are more distinctive from each other, making them easier to remember (Nelson et al., 1976). There are pieces of evidence supporting each of these views, and it seems likely that each accounts for some part of the consistent superiority of pictures and visual stimuli over the verbal stimuli that memory researchers frequently use as a matter of convenience. The critical point is that visual long-term memory appears to be better than verbal long-term memory. This should leap out to students in emphasizing the importance of studying the figures in the articles of *Attention, Perception & Psychophysics*, instead of focusing your learning on these words.

### The biological basis of memory

How the neurons of the visual system form the memories we recall of the events from a fraction of a second ago, yesterday, or our last birthday is critical for our understanding of the principles of memory that we have discussed in this article so far. Given our discussion of the different types of memory storage, you might also expect that different types of neural activity and their structure support these distinct memory types.

**Fig. 9:**
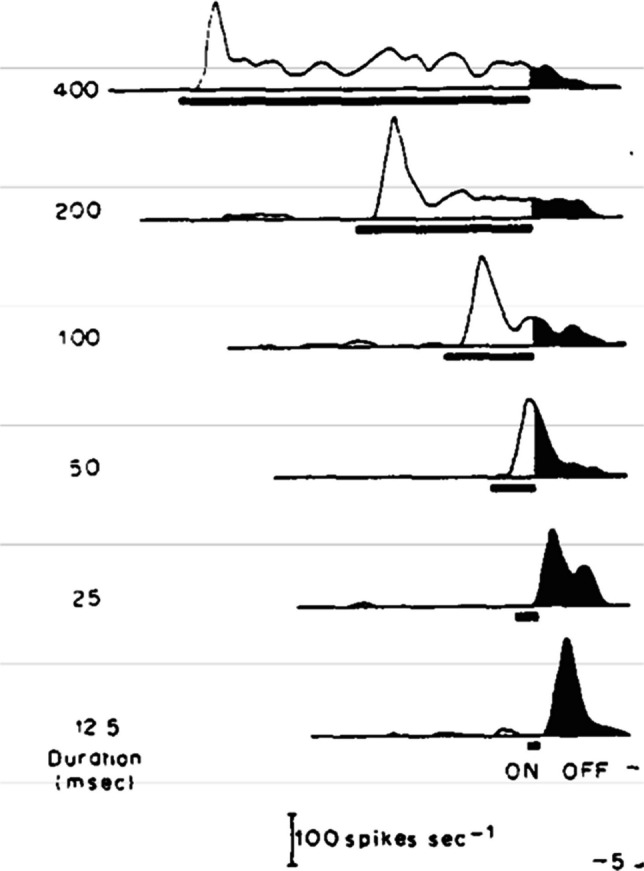
This figure shows the lingering visual response in cat area 17 to stimuli of varying durations. These plots show that the duration of the after effect is inversely related to the duration of the stimulus (i.e., the after effect is smaller when the stimulus is on for longer, just as we experience with these sensory memory effects). From Duysens et al. (1985)

Iconic memory is the brief, high-capacity storage of a scene that has just gone out of view. This information appears to be stored as the residual activity of the visual neurons that code for the sensory input (Irwin & Thomas, 2008). After a stimulus is flashed, that information leaves a lingering trace in neurons throughout the visual system, from the retina to the frontal lobe. As shown in the recordings of a cortical brain region of the cat, known to process visual information (area 17) in Fig. 9, and this lingering response is extended in time as the visual input propagates through the visual hierarchy of areas (Schmolesky et al., 1998). This lingering visual response has been linked to the iconic memory representation of the stimulus both with its time course and its sensitivity to the contrast of the eliciting stimulus relative to the environment (Duysens et al., 1985). These action potentials that continue as the activity of the neurons return to baseline appears to support our quickly fading iconic memory.

Visual working memory is believed to rely on a different type of neural activity. Specifically, theories of memory storage have long proposed that the temporary maintenance of memory representations relies on the reverberations of the network of neurons that code for the objects and scenes that we store in visual working memory (Hebb, 1949). The representations of objects in the brain are distributed across large networks with different selectivities (DiCarlo et al., 2012). Let us consider an example in which you remember Ernie from *Sesame Street* (Tsunoda et al., 2001). We believe all of the neurons coding for Ernie’s visual features would fire in a concerted way when you store an instance of Ernie into visual memory or retrieve an instance from memory. This type of concerted, cyclical activity is referred to as oscillatory or reverberatory. As we hold the image of Ernie active in visual working memory, the network of neurons should exhibit pulses of activity that rise and fall at the same time. It is believed that these temporally linked pulses of activity form the unified representations of objects that we experience in working memory (Vogel et al., 2001). In addition, the theory that these networks of neurons firing in a concerted way, at the same time, means that they are likely to form long-term memory representations.

The creation of long-term memory representations is believed to be due to neurons becoming wired together (Antonov et al., 2003; Hebb, 1949). When one neuron in the visual system fires action potentials at the same time as another neuron that it is connected to, the synaptic connections between those neurons are strengthened. The program of research focused on understanding the structural and chemical changes that strengthen those connections is vast (Hawkins et al., 2006; Kandel & Tauc, 1965). A complete understanding of this plasticity of the synaptic connections between neurons, and the cascade of protein synthesis necessary for such plasticity is beyond the scope of this introductory tutorial. For our purposes, it is sufficient to know that cells that fire together wire together (Löwell & Singer, 1992). It is believed that long-term memories are stored in the wiring together of neurons that were simultaneously active when we viewed a scene or a face or experienced an event that we can later remember. The idea that visual working memories are maintained by keeping the networks of neurons simultaneously active are precisely the conditions that would result in those cells becoming wired together and underlies the idea that representing information in visual working memory is key to forming long-term memories of that same information (Hebb, 1949). Because long-term memory is stored in these connections between neurons this means that our long-term memories are not stored in one place in the brain, instead, they are due to a distributed pattern of activity across multiple neurons (Lashley, 1950). This distributed nature of our long-term visual memories can help explain several key features of our memories.

By storing our visual memories in the connections across neurons, we can avoid the dangers of a localist representations scheme (Bowers, 2002). A localist representation is when we store a memory in a single neuron (or even a very small network of a few neurons). For example, if you stored your memory of your grandmother’s face in one neuron, then if that one neuron was lost due to the natural process of cell death or a heavy night of celebration at a pub, then you would have no idea what your grandmother’s face looked like. As evidence against this idea, our brains demonstrate *graceful* *degradation* (Nadeau, 2020), in which the loss of a memory, skill, or ability degrades slowly across long time scales, not suddenly, as would be the case if memories were stored in individual neurons or small pools of a few neurons.

The network nature of memory representations can be observed in a variety of ways. One way to verify that memories are stored across a broad network of neural activity is to watch this activity spread across the network. Using functional magnetic imaging, researchers have shown that when I ask you to recall a particular face that you previously saw, that the same broad network of activity is measured as you recall that face as when you encoded it during a study phase of the experiment. When we want to retrieve our memories of things that we have viewed in the past, it appears that we do this by reactivating the network that was active when we saw that stimulus previously (Polyn et al., 2005). This process of internal reactivation continues to be studied, but we will discuss how we think this occurs shortly.

The effects of the network connections underlying our long-term memories can be measured in the laboratory using priming paradigms. You can imagine that if you have a network of neurons in your visual system that store your memories of dogs, then that network will be more similar to the network that stores our memories of what cats look like than the network that stores what furniture looks like. That is, the networks representing two objects that are visually similar will be highly overlapping in terms of the neurons that they activate. This means that if you could activate one of these networks, then some residual activity in that network may linger, making it easier to activate the network needed to process the new stimulus. Say we show you the word ‘butter’, with this presentation resulting in you activating your network representation of butter (i.e., what it looks like, how it is spread, maybe what it tastes and smells like, etc.). Shortly after that word, we show you a picture of a dog, and you have to name the animal shown in that picture—that is, say “dog.” We can imagine that the network of connections that represents “butter” in long-term memory will be very different from the one that represents “dog.” On another trial we present the word “cat,” followed by a picture of a dog to be named. In this second case, if the networks for cat and dog overlap more than the networks for butter and dog, then people should be faster to say “dog” when preceded by “cat.” This is exactly the type of result that has been observed across many semantic priming experiments (Reinitz et al., 1989).

Semantic priming is even found when you present a picture of an object so briefly that people cannot report that anything was actually shown (Dell'Acqua & Grainger, 1999). This suggests that even a little activity in a network that is below our level of awareness is sufficient to increase the efficiency of activating the network necessary to access information stored in long-term memory. These appear to be automatic effects in which memory representations feed activity to one another. Next, we will talk about the neural basis of trying to remember something that we have previously stored in the connections across networks of neurons.

### The medial-temporal lobe and hippocampus

We owe much of what we know about how long-term memory works to patients what have suffered trauma to their brains (Corkin, 1968; Graf et al., 1984; Milner et al., 1998). The single most important patient who changed what we know about memory was known by his initials, H.M., though his name, Henry Molaison, was made public after his death (Corkin, 2013; Squire, 2009). H.M. suffered from a case of epilepsy that became impossible to treat with the pharmaceuticals available in 1958. The seizures appeared to originate in the medial temporal lobe, so the decision was made by his neurosurgeon, William Scoville, to bilaterally remove large portions of this brain region, including a region known as the hippocampus. The surgery was successful in eliminating H.M.’s seizures. However, it also removed H.M.’s ability to form new episodic memories of his ongoing experience, a condition known as anterograde amnesia. His case demonstrated the importance of the hippocampus and surrounding medial temporal lobe cortex for episodic memory. Furthermore, by demonstrating that other kinds of memory were more or less intact (e.g., procedural memory), his case provided support for the idea that the human brain has multiple, functionally independent memory systems. In the years since H.M.’s surgery, we have learned a great deal more about how the structures of the medial-temporal lobe support the formation and retrieval of memories.

The hippocampus appears to work like the social media network for the brain, linking together the neurons that are active during a learning event (McClelland et al., 1995; Squire et al., 2004). Let us consider the example of being at a political campaign event, like the one shown in Fig. 1. Being at that event will stimulate neurons across a huge number of visual areas of the brain (V1, V2, V4, IT, face-selective neurons, etc.), but also neurons elicited by the sounds, smells, those activated by thinking about abstract concepts like hope or change, just to name a few of the features of the event. The constellation of cortical brain activity representing all the features of an event seem to be linked to one another with the support of the hippocampus. After being formed, it seems that an event memory like this is dependent on the hippocampus to be retrieved (Tse et al., 2007). However, with the passage of time, memories gradually become less dependent on the hippocampus for retrieval, possibly because the cortical components of the memory become associated to one another, no longer requiring the hippocampus to reactivate the entire ensemble of features (Moscovitch et al., 2016).

The hippocampus is directly connected to area V1 and other visual areas (Markov et al., 2014), allowing it to support the reactivation of visual neurons while remembering a past experience. When we remember what the picture in Fig. 1 looked like tomorrow, the hippocampus will support the reactivation of the network of neurons initially stimulated by the photons reflected off of the picture of the election event.

### Models of visual memory

We began this article by discussing how the modal model of memory guided many explorations of human visual memory (Atkinson & Shiffrin, 1968). Now, we are returning to models of human memory to describe the diversity of mechanisms that have been proposed to account for performance in specific tasks. True to the spirit of the comprehensive model of human memory, we discuss general models of human memory which can handle visual information, not just models designed to account for data from visual memory experiments. We also note that this section of our review shifts our focus in depth. That is, we will discuss how the detailed mechanisms of the models result in broad patterns of human-like behavior that we have reviewed up to this point. That said, a comprehensive and technical treatment is beyond the scope of this tutorial. However, many excellent resources exist to give the interested reader a deeper understanding of computational models of memory (Kahana, 2012, 2020; Malmberg et al., 2019; Oberauer, 2023; Raaijmakers & Shiffrin, 1992).

Signal detection theory is a modeling framework used to understand memory-guided decisions (Green & Swets, 1966; Kintsch, 1967; Macmillan & Creelman, 1991; Murdock, 1965). In a recognition task, a probe is presented, and a participant is asked whether they observed that probe during an earlier study period. The probe representation is projected into the memory system, which returns a strength signal. However, this signal is noisy. That is, the signal contains random, unpredictable variability. Random noise is recognized widely in cognitive modeling as a useful component of cognitive processing, rather than a nuisance to be minimized (Farrell & Lewandowsky, 2018; Luce, 1986; Wiener, 1948). Here, noise adds variability to a participant’s responses, which helps the model capture variability in human performance.

**Fig. 10:**
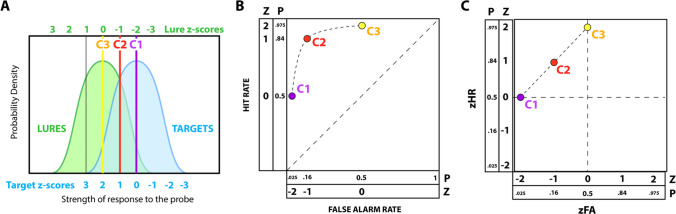
A schematic representation of signal detection theory. **A** Each test probe elicits a response from the memory system, with target probes producing on average stronger responses than lure probes. This schematic depicts an equal-variance signal detection model, where target and lure strength distributions have the same shape. Because the distribution of strength values elicited by targets and lures overlap, perfect performance is impossible. A threshold (C1, C2, or C3) must be used to decide whether to respond that a particular probe was studied. **B** Each potential threshold produces a point on a receiver operating characteristic (ROC) plot, where the axes reflect the probabilities of making hits and false alarms. C1 is a conservative threshold, causing the participant to miss half of the target probes, but producing very few false alarms. C3 is a liberal threshold, allowing the participant to correctly identify nearly all target probes but producing false alarms for half of the lure probes. **C** Hit rate and false-alarm rate can be expressed in terms of z-scores relative to the hypothetical strength distributions from panel A, producing a zROC plot. When this transformation is applied to an equal-variance signal detection model, the performance associated with different thresholds traces a line with a slope of 1. In contrast, data from human participants tends to have a slope of less than 1 on a zROC plot, which supports an unequal-variance model

The strength values produced by things you studied (targets) are generally stronger than the strength values produced by things you did not study (lures). Because of the above-mentioned random noise, there is variability in these strength distributions, and this variability is described using a probability distribution, as visualized in Fig. 10A. In other words, the strength distributions for targets and lures overlap, so there is no way to perfectly discriminate one from the other (Kintsch, 1967). The best you can do is pick a particular decision threshold and accept that you will get a mix of hits (correctly identifying a target), misses (failing to identify a target), false alarms (erroneously accepting a lure as studied), and correct rejections (correctly rejecting a lure as not studied).

If a participant provides an estimate of confidence or certainty in their response, a researcher can perform a receiver operating characteristic (ROC) analysis to determine the sensitivity of the participant to discriminate targets and lures (Yonelinas & Parks, 2007). Signal detection theory can be used to infer certain characteristics of the distribution of strength values for targets and lures from the distribution of points on an ROC plot. If the hit rates and false-alarm rates are converted using a *z*-transformation, many recognition memory studies produce a linear set of ROC points with a slope of less than one. This indicates that the strength distribution for target items is stretched out relative to the lure distribution, which is usually taken as evidence that there is variability in the efficacy of learning from item to item (Kahana, 2012).

Signal detection theory is a powerful framework that has been used to understand how the distributional properties of memory strength relate to memory-guided decisions, how the conditions of retrieval affect memory performance (Annis et al., 2013; Criss et al., 2011), and has informed or been incorporated into many types of computational models of memory (Osth & Dennis, 2015; Schurgin et al., 2021; Shiffrin & Steyvers, 1997). One of the powers of signal detection theory is the abstract way it characterizes the memory system. However, this abstraction makes signal detection theory less suitable for certain aspects of memory theory, such as the structure of memory representations and the nature of the learning process. For insight into these aspects of memory, we turn to cognitive process models.

Many cognitive theories propose that every experience is encoded as a representation and has the potential to form an enduring memory trace (Polyn, 2024). Different modeling frameworks approach this with different levels of abstraction. Connectionist models treat representations as patterns of activation across populations of elements with dynamics inspired by neurons (O'Reilly & Munakata, 2000; Rumelhart & McClelland, 1986). Neurons communicate through networks of synaptic connections, and memory formation involves adjusting the strength of these connections (Dayan & Abbott, 2001). The webs of connections between neurons can store many memory traces. A probe representation that is similar to a particular past experience can retrieve the details of that experience by allowing the activation dynamics of the neural network to reactivate the missing features (Hopfield, 1982; McClelland et al., 1995).

Instance-based models treat memory formation and retrieval abstractly and have deep similarities to the connectionist approach (Jamieson et al., 2022). Each experience is encoded as a representation, and each of these representations is added to an ever-growing stack of memory traces (as in MINERVA;Hintzman, 1986). A probe representation that is similar to a particular past experience is compared to the stack of memory traces, and the system returns a strength value indicating how well the probe matches the stored traces (allowing simulation of recognition tasks) or returns an echo representation that reactivates the missing features from the stored traces (allowing simulation of cued recall and free-recall tasks).

Instance models like the generalized context model (GCM) explain how core cognitive abilities such as discrimination, identification, recognition, and categorization arise from the vast number of memory traces created through our interactions with the objects that populate our everyday experience (Nosofsky, 1987, 1992). A noisy-exemplar model (NEMO) builds upon GCM and MINERVA to account for human performance in short-term visual recognition tasks (Kahana et al., 2007). In these tasks, a memory probe that partially matches several study items often produces an erroneous endorsement of this item as having been studied (a false alarm). This effect is well-known in studies of verbal memory. Kahana and colleagues demonstrated that the same effect is seen with hard-to-verbalize visual study items (compound spatial gratings) where item-to-item visual similarity can be precisely specified in terms of the spatial frequency of the gratings (Danker et al., 2008; Kahana et al., 2007). NEMO explains these false-alarm errors as arising from a summed-similarity mechanism (common to many instance-based models), whereby signals from several partial matches between the probe representation and the stored memories are added together. If this aggregated signal crosses a critical threshold, the model triggers a positive response that the item has been seen before, even if the probe item was not on the study list.

The instance theory of attention and memory (Logan, 2002) incorporates aspects of several of the above-described models and integrates them with computational models of attention (e.g., the theory of visual attention; Bundesen, 1990). ITAM characterizes the deep relationship between attentional processes and memory processes. If you are looking for your child in a crowded school corridor, attentional processes allow you to efficiently scan the small faces in the crowd. ITAM describes how categorization processes support this attentional deployment by guiding your gaze to the children’s faces as opposed to other visual features of the corridor, and how memory processes are engaged to identify the face of your own child once it is fixated.

One of the most reliable principles of human memory is the law of recency, the idea that all else being equal, recent events are more accessible than more distant events (Kahana, 2012). Many visual models account for the law of recency with a decay explanation. As described above, performance limitations in iconic memory tasks are explained by a representation that fades away rapidly as time passes. However, another class of memory models propose that certain recency time-based effects arise from the dynamics of an internal representation of temporal context. These models focus on characterizing the dynamics of memory search in verbal memory tasks like serial recall and free recall (Kahana, 2020). In these tasks, a participant studies a series of items and then reports the item identities verbally (either by speaking or by typing). As such, these tasks may seem to have questionable relevance to our understanding of visual memory. However, computational models of these memory tasks provide insight into the cognitive mechanisms at play in both verbal and nonverbal memory tasks.

According to retrieved-context models, every event memory has two components: First, the features of the people, places, and things comprising the event, and second, a gradually evolving contextual timestamp indicating the temporal context of the event (Polyn & Kahana, 2008). Retrieved-context models propose that this temporal context representation is used to target memories, and because the state of temporal context changes gradually, the current timestamp is a good cue for recently formed memories, allowing the model to capture recency effects (Sederberg et al., 2008). This timestamp imbues the memory system with another power, that of mental time travel (Tulving, 2002). The idea here is that when a memory from further back in time is retrieved, that memory’s timestamp is also retrieved. When this recovered timestamp is incorporated into one’s retrieval cue, this makes other past events that happened nearby in time to that memory to become more accessible, as if one is revisiting their past experience.

**Fig. 11:**
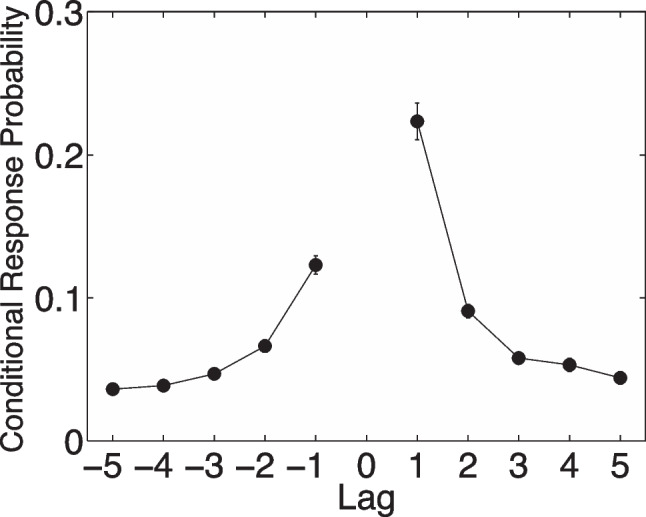
A lag-based conditional response probability plot showing the likelihood of recalling an item as a function of its order in a list. The 0 lag is the current item recalled, and the flanking probabilities show the likelihood of recalling the neighboring items in the temporal stream

In the free-recall task, participants report a set of studied items in whatever order they come to mind. The order in which the items are remembered provides insight into the organizational structure of memories—that is, how retrieving one memory can prompt you to remember other, related memories (Puff, 1979). After retrieving one item, the most likely next response is an item from a neighboring list position (see Fig. 11), further demonstrating that the temporal structure of a study experience is embedded in the memories for the individual details of that experience. According to these models, temporal organization of memories arises from the memory cuing process described by these models. When an item is remembered, its temporal contextual timestamp is also reactivated. The retrieved temporal context is incorporated into the probe representation used to guide further memory retrieval, making memories of nearby points in time more accessible.

This integrative temporal context mechanism, developed to explain recall dynamics in a verbal list-learning task, provides insight into the rapid and complex sequence of eye movements seen during a visual scene recognition task (Kragel & Voss, 2021). Kragel and Voss (2021) examined several eye-tracking memory experiments to demonstrate that the sequence of eye movements (the scan path) elicited by a visual scene is recapitulated when that scene is observed again, in a way that cannot be explained by the low-level properties of the stimulus. Furthermore, the degree of scan path reinstatement predicted successful memory for the scene. In general, the temporal context mechanism is a useful theoretical tool for understanding memory for experiences that unfold over time.

At the beginning of this review, we discussed how performance in the short-term change-detection task demonstrates that only a handful of items from a briefly presented display are successfully remembered when a probe is presented a few seconds later. These performance limitations are explained by modern computational implementations of the modal model in which visual working memory is characterized as consisting of a small set of slots, each of which can store one item representation. When the number of items in a visual array exceeds the number of slots, only some items are stored, and the others are forgotten. If a probed item is stored in a slot, the model predicts a correct response; if the probed item is not in a slot, the model uses a guessing process to make the response. Resource-based models provide an alternative account for these performance limitations. In these models, capacity is conceptualized as a limited cognitive resource that is spread thin when the study items are too numerous. These resource-based models have been implemented using the signal detection framework we reviewed above, as signal detection theory provides a natural way to account for memories with variable strength (Schurgin et al., 2021; Williams et al., 2022).

With a computational modeling approach, different theories of visual working memory structure are reified as different computational models, which can then be put head-to-head to compare their ability to account for key phenomena in visual working memory tasks. Despite the general dominance of slot-based models in modern visual memory theory, modeling studies do not provide a definitive answer as to which model type captures human performance better. In many studies, slot-based models match participant performance better than resource models (e.g., Donkin et al., 2013, Rouder et al., 2011). However, other studies show mixed results (Donkin et al., 2014), inconsistencies in the predictions of the slot-based models (Bays, 2018), or consistent support for resource-based models (Robinson et al., 2020, 2025; Williams et al., 2022). It is perhaps not surprising that modeling studies produce a diversity of results, as there is great methodological heterogeneity across studies of visual working memory. Many aspects of experimental design (e.g., type of memoranda, kind of memory test, distribution of trial types within a session) and participant response strategies affect performance, which in turn affects model fitness in ways that are difficult to untangle.

Despite the theoretical focus on head-to-head comparisons between slot-based and resource-based models of visual working memory, there are other modeling approaches that provide important insight into the cognitive machinery supporting performance in these tasks. The Interference Model describes performance limitations as arising from interference between recently formed memories (Oberauer & Lin, 2017), and the Context Maintenance and Retrieval-Working Memory (CMR-WM) model describes performance limitations as arising from a temporal context-based targeting mechanism similar to the one that guides free-recall performance (Polyn & Woodman, 2025). The Target Confusability Competition (TCC) model explains performance limitations as arising from the nonlinear mechanism that computes the similarity between a potentially noisy retrieved memory and the different choice alternatives available at test (Schurgin et al., 2021). TCC demonstrates that by carefully measuring the psychophysical similarity of the studied items, and incorporating these similarity estimates into the model, many important aspects of human performance, such as the fine-grained shape of the response error distribution in a continuous report visual working memory task, can be captured with only a single free parameter.

The connectionist researchers of the 1980 s developed neural network models that demonstrated how basic neuroscientific principles such as neuronal activation and synaptic communication can be used to guide theory development (McClelland et al., 1986). The HMAX model of object recognition used these neural networks to explain how people’s long-term memory for the visual forms of objects guides their performance in object recognition tasks (Riesenhuber & Poggio, 1999). More recently, scientists discovered that these connectionist models could be supercharged by pairing error-driven synaptic learning rules with deep architectures containing many representational layers, allowing the models to perform impressive tasks, such as identifying thousands of categories of objects in natural pictures (Krizhevsky et al., 2017; LeCun et al., 2015). For the most part, these models were not developed to account for human learning and memory (but see Hedayati et al., 2022), but they do produce human-level performance on basic object recognition tasks and have been used as models of neural responses in the human visual system (Kriegeskorte et al., 2015).

Modern artificial intelligence algorithms provide new perspectives into the structure of human language that have been incorporated into modern models of the human visual system. These language models assign a representational vector to each word in a language, and the similarity of these semantic vectors reflect the relatedness of the corresponding words (Lenci et al., 2018). The semantic salience model uses these semantic vectors to represent the structured long-term semantic memories a person has of the world and the things in it (Hayes & Henderson, 2019). Using these vectors, the model captures the deployment of attention in real-world scenes, including enhanced attention given to regions of a scene containing semantically related objects (Hayes & Henderson, 2022; Table 1).

**Table 1:** Overview of models named in the text

Model name	Description	Representative Publication
REM: Retrieving Efficiently from Memory	Instance-based model of recognition memory	Shiffrin and Steyvers, 1997 (PB&R)
GCM: Generalized Context Model	Instance-based model of categorization and memory	Nosofsky, 1986 (JEP:G)
NEMO: Noisy Exemplar Model	Instance-based model of visual short-term memory	Kahana et al., 2007
MINERVA	Instance-based model of memory	Hintzman, 1984 (Behavior Research Methods)
Interference model of working memory	Model of visual working memory capacity due to interference between representations	Oberauer and Lin, 2017, 2023
ITAM: Instance Theory of Attention and Memory	Instance-based model integrating many theories of attention and memory	Logan, 2002
TCM: Temporal Context Model	Neural network model of memory search	Howard and Kahana, 2002
TCC: Target Confusability Competition	Decision noise model of choices	Schurgin et al., 2021
CMR: Context Maintenance and Retrieval	Neural network model of memory search incorporating source and semantic context	Polyn et al., 2009 (Psych review)
CMR-WM: Context Maintenance and Retrieval-Working Memory	Instance-based model of visual working memory	Polyn and Woodman, 2025
HMAX	Neural network model of object recognition	Riesenhuber and Poggio, 1999
Semantic salience model	Vector-space model of semantic influences on visual attention	Hayes and Henderson, 2019

### New directions

Instead of providing an exhaustive list of unresolved issues related to visual memory we highlight two thriving questions in the current literature. The first question is the big one. How is working memory related to long-term memory? This is not a new question. It was the same one that William James began with when considering the nature of primary and secondary memory systems (James, 1890). The nature of this relationship has grown in importance given the proliferation of neuroscientific measures of human memory (Woodman et al., 2022). These brain measures allow us to peer under the hood of the human mind and give insight into the nature of memory storage in the brain. We believe that the next generation of cognitive neuroscientific computational models may unify theories of working memory and long-term memory, allowing us to understand how common cognitive machinery supports the flexible use of memory at both shorter and longer timescales (Polyn & Woodman, 2025).

A second issue that Psychonomes are vigorously seeking answers to is whether long-term memory storage modulates or even determines what objects are selected by attention mechanisms. Approximately a decade ago, several laboratories simultaneously began focusing on the question of whether information acquired across the long term can bias attention during the processing of complex visual scenes or listening in noisy environments (Awh et al., 2012; Carlisle et al., 2011; Zhao et al., 2013). These views grew out of classic work showing learning effects during attention-demanding tasks (Chun & Jiang, 1998; Logan, 1978; Maljkovic & Nakayama, 1994). It is beyond doubt that the accrual of information across trials is changing how we deploy attention (B. A. Anderson et al., 2021). Moreover, leading theories of learning and automaticity propose that such effects are a natural result of storing each task episode (Logan, 1988, 2002; Rickard, 1997). Given that the power functions that describe learning curves are modeled by all of our memories racing for retrieval when we encounter a new stimulus array (Logan, 2002), then these models predict just the patterns of findings that researchers have consistently obtained in which information embedded in arrays across trials come to bias attention in future arrays (Anderson et al., 2021).

### Summary

Visual memory is remarkable in its severe limitations observed over the span of a few seconds and its essentially unlimited storage when viewed across the lifespan. Our ability to recognize and recall visual information is as close as humans have to perfect memory for any type of information that we can store. Here, we provided an overview of where we are now and how we got here, from the modal theoretical perspective in cognitive psychology. This appears to be a critical time to be studying visual memory as it has taken a central place in helping us to study basic brain functions. This is logical as it capitalizes on the status of the visual system as the model sensory system, with our unprecedented knowledge about neural activity and its wiring across brain and within brain areas (Kaas, 2005).

## Data Availability

Not applicable.
